# Pulmonary tuberculosis: a differential diagnostic priority in unprovoked deep venous thrombosis patients with haemoptysis

**DOI:** 10.11604/pamj.2018.29.57.14225

**Published:** 2018-01-22

**Authors:** Nahid Azdaki, Seyedali Moezi, Marjan Farzad

**Affiliations:** 1 Cardiovascular Diseases Research Center, Birjand University of Medical Sciences, Birjand, Iran

**Keywords:** Deep vein thrombosis, haemoptysis, pulmonary tuberculosis

## Abstract

Deep vein thrombosis (DVT) is a common cause of death worldwide. Several factors are associated with increased risk of DVT. In this report a case of deep venous thrombosis (DVT) of the lower limb and its link with underlying pulmonary tuberculosis is described in a young male patient with haemoptysis.

## Introduction

Venous thromboembolism (VTE) is a preventable and curable cause of death worldwide. There are several factors that are associated with increased risk of VTE such as age, major surgeries, prolonged bed rest, oral contraceptive pills, malignancy, central venous line, smoking and inherited hypercoagulable states [1]. Tuberculosis and its thrombogenic potential can be a neglected risk factor for deep venous thrombosis [2]. In the literature, a rare but dangerous linkage between tuberculosis and Deep vein thrombosis has been reported [3-5]. In some cases, the symptoms patients are encountered while DVT treatments or even at the time of admission to the hospital are related to such underlying causes which firstly may not be noticeable. That is why it is important to sensitize all clinicians to this matter by reporting a case observed in our hospital.

## Patient and observation

**Figure 1:**
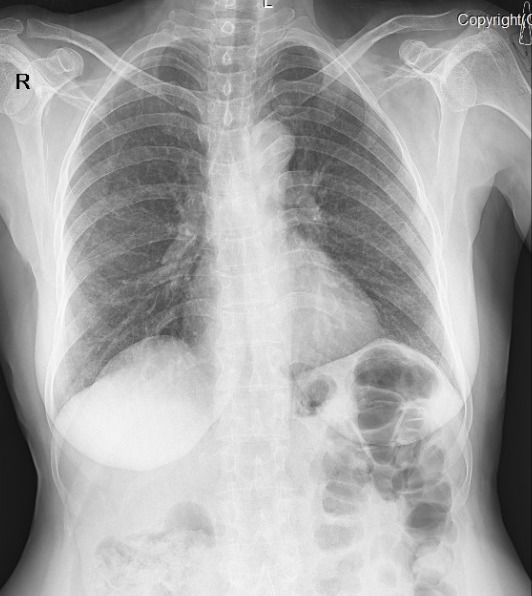
The patient’s chest X-ray

**Figure 2:**
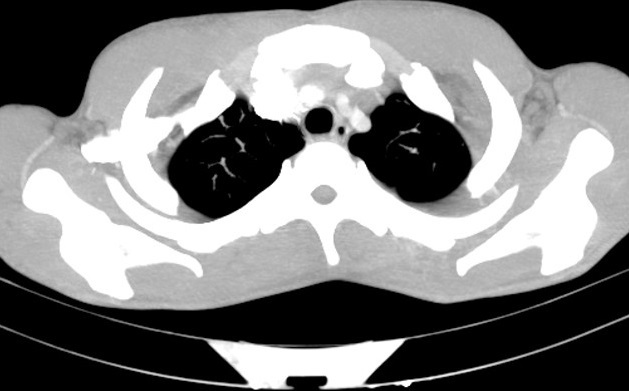
The patient’s CT pulmonary angiography

A 22-year-old male was admitted to Valiasr Hospital affiliated to Birjand University of Medical Sciences Iran, with complaints of painful swelling of the left lower limb for the last two days. His vital signs were stable (oral temperature 37.3^o^C, pulse rate = 67, respiratory rate = 16, systolic blood pressure = 120 and diastolic blood pressure = 80) and his physical examinations, except, the swallowed tender left leg (from ankle up to the thigh) were unremarkable. Chest-x-ray, abdominal sonography and echocardiography were normal. CBC revealed mild normocytic anemia (Hb: 11 gr/dl) with normal WBC and Plt count (6.4, 172*103 respectively). The erythrocyte sedimentation rate and the c reactive protein were elevated unexpectedly (ESR = 50 mm/h, CRP = >10mg/L). Routine biochemical investigations, thyroid function tests and tumor markers were normal. He had neither undergone any major surgeries in recent past nor history of prolonged immobilization, other diseases and smoking. Ultrasound Doppler study of the left lower limb revealed DVT in left popliteal up to external iliac veins. The patient was treated through catheter directed thrombolysis (CDT) with infusion of streptokinase 250.000 unit stat and 100.000 unit per hour infusion for 48 hours with concomitant 500 unit per hour of heparin and taking warfarin tablets 5mg/daily. During hospitalization he had severe pleuritic chest pain that we thought that is due to small distal pulmonary embolism which is a common finding in venous thromboembolism. The patient discharged from hospital with a good general condition after 10 days with international normalized ratios (INR = 2), partial thromboplastin time (PTT = 30) and Prothrombin Time (PT = 18.4). A high sedimentation rate (ESR: 50) was our question mark at the patient discharge time when all primary evaluations were normal. The day after discharge, the patient re-admitted with cough, haemoptysis and pleuritic chest pain. The patient had blood -streaked sputum 6 times a day which was not massive (10 cc/24h). Laboratory tests showed an elevated ESR (80 mm/h) and CRP (139 mg/l). There was hemoglobin decline (hgb = 10.5 gr/dl). Biochemical findings were within normal limits. CXR and pulmonary CT angiography showed minimal pleural effusion and massive PTE and pulmonary necrosis were ruled out (Figure 1, Figure 2). Along with His subjective and objective findings a clinical suspicion to TB was raised that a surprising positive result of the sputum for acid fast bacilli (3 samples) confirmed it. Since the patient's symptoms were in the context of pulmonary tuberculosis, not lung infarction, he was referred to infectious service for TB treatment.

## Discussion

Deep vein thrombosis (DVT) and pulmonary embolism (PE) are the two manifestations of venous thromboembolism (VTE). It is not uncommon to think of a pulmonary embolism in a DVT patient with haemoptysis. There are several major causes of haemoptysis such as infections (mycobacteria, fungal infections), neoplasm, iatrogenic/truma, pulmonary infarct/embolism, coagulopathies, cardiovascular, vascular and vasculitis. Tuberculosis is still the most common cause of haemoptysis worldwide [6]. Deep venous thrombosis has been associated with 1.5% to 3.4% cases of tuberculosis (TB). 25% to 50% of patients with first-time DVT have no identifiable factors and its association with TB is rare or commonly associated with advanced stages of tuberculosis [7]. In this sense there are many case reports of DVT associated with tuberculosis [8-12]. Gathwala et al. (2016) presented a case of DVT in a 13-year-old female as a complication of childhood abdominal tuberculosis [13]. To the best our knowledge severe TB is known to induce an acute phase response which leads to the activation of mononuclear cells; the interaction of mycobacterial products with activated mononuclear cells subsequently induces increased synthesis of tumour necrosis factor-α and interleukin. These proinflammatory cytokines activate the vascular *intima*, rendering the endothelial surface thrombogenic. The cytokines also induce a hepatic acute phase response leading to deranged levels of coagulation proteins and a subsequent hypercoagulable state [14]. Noteworthy, the absence of cavitary lesions from TB in our patient's chest x-ray and other TB symptoms such as night sweats, anorexia and weight loss caused attention was paid to the most likely diagnosis for a DVT patient with haemoptysis, that is, *pulmonary embolism*, although some diagnostic changes, sign or symptoms may appear in severe or more advanced stages of the disease. It is not exactly clear whether DVT in our case is associated with TB or not but what matter is paying attention to this differential diagnosis along with other possibilities in confronting with a DVT patient with hemoptysis, especially, in patients with similar symptoms who live in endemic TB areas.

## Conclusion

Our case highlights the importance of a high index of suspicion of pulmonary TB in DVT patients. Pulmonary tuberculosis should be considered as a differential diagnostic priority in unprovoked deep venous thrombosis patients with haemoptysis, especially in patients live in endemic TB areas.

## Competing interests

The authors declare no competing interests.
